# The vermian-crest angle: a prediction model for foetal cystic posterior fossa anomalies

**DOI:** 10.3389/fnins.2025.1623105

**Published:** 2025-09-17

**Authors:** Marialuigia Spinelli, Sofia Amylidi-Mohr, Daniel Surbek, Kai-Sven Heling, Matthias Scheier, Leo Pomar, David Baud, Luigi Raio

**Affiliations:** 1 Department of Obstetrics and Gynecology, University Hospital Bern, University of Bern, Bern, Switzerland; 2 Centre for Prenatal Diagnosis, Berlin, Germany; 3 Foetal Medicine Service, Outpatient Department for Foetal Medicine, Feldkirch, Austria; 4 Ultrasound and Foetal Medicine Unit, Department Woman-Mother-Child, Lausanne University Hospital, Lausanne University, Lausanne, Switzerland

**Keywords:** vermian-crest angle, posterior fossa, vermis, cerebellum, prenatal diagnosis, three-dimensional ultrasound scan, Blake’s pouch cyst, Dandy–Walker malformation

## Abstract

**Background:**

Accurate categorisation of the upward rotation of the foetal cerebellar vermis continues to pose diagnostic challenges in prenatal medicine. Recently, a new parameter of the posterior fossa (PF), known as the vermian-crest angle (VCA), has been evaluated using three-dimensional ultrasound (3D-US) and prenatal magnetic resonance imaging (MRI).

**Objective:**

This study aimed to evaluate the performance of the VCA in categorizing PF anomalies using 3D-US and to determine its level of agreement with MRI.

**Study design:**

We conducted a cohort study involving confirmed cases of PF anomalies. We measured the VCA using 3D-US and compared our data with previously published reference values obtained through both 3D-US and MRI. For statistical analysis, we employed univariate analysis of variance (ANOVA) followed by Tukey’s post-hoc test, receiver operating characteristic (ROC) curve analysis, and the intraclass correlation coefficient (ICC).

**Results:**

We identified 53 foetuses at a mean gestational age (GA) of 24.5 (SD 5.45) weeks with Blake’s pouch cyst (BPC) (*n* = 11), Dandy–Walker malformation (DWM) (*n* = 9), mega cisterna magna (MCM) (*n* = 22), and vermian hypoplasia (VH) (*n* = 11). Compared to published reference values, the VCA was significantly increased in DWMs (mean 130.6°, SD 16.75°; *p* ≤ 0.01) and BPCs (mean 91.00°, SD 19.73°; *p* ≤ 0.05). A VCA > 80.1° distinguished BPCs and DWMs from other PF anomalies, while a VCA > 107° differentiated BPCs from DWMs. When comparing subgroups with published MRI data, we found good agreement between 3D-US and MRI (ICC = 0.71) (95% CI: 0.55–0.87).

**Conclusion:**

The VCA may be helpful in categorising PF anomalies using 3D-US, particularly BPC and DWM. The good agreement with MRI measurements reinforces the synergy of these tools in the diagnostic work-up.

## Introduction

Imaging of the foetal posterior fossa (PF) is currently an integral part of neurosonography, particularly in cases where anomalies are suspected (Zalel et al., 2006; Milani et al., 2019; Limperopoulos et al., 2006; Barkovich et al., 2009; Robinson, 2014; Tilea et al., 2007). Normal sonographic (Ultrasound, US) biometry and morphology of the PF can effectively rule out most major anomalies of the foetal cerebellum and vermis (Pertl et al., 2019). Nevertheless, when the PF appears abnormal, differential diagnosis remains challenging, with possibilities ranging from benign, asymptomatic conditions to severe malformations associated with significant neurological impairments (Limperopoulos et al., 2006; Barkovich et al., 2009; Robinson, 2014; Tilea et al., 2007; Pertl et al., 2019; Vatansever et al., 2013; ten Donkelaar et al., 2014; Kapur et al., 2009; Dekain et al., 2014; Poretti et al., 2014; Barzegar et al., 2013; Xie et al., 2001; Zalel et al., 2002; Rizzo et al., 2012; Viñals et al., 2005; Malinger et al., 2001; Altmann et al., 2020; Lei et al., 2015; Altmann et al., 2020; Paladini et al., 2019; Sun et al., 2019). For example, the four most common anomalies—Dandy–Walker malformation (DWM), vermian hypoplasia (VH), Blake’s pouch cyst (BPC), and mega cisterna magna (MCM)—often share similar sonographic features during screening, despite having markedly different prognoses (Katorza et al., 2016). In the majority of PF abnormalities, there is typically a wide communication between the fourth ventricle and the PF, along with a reduced size of the vermis. However, according to a recent classification (Robinson et al., 2007), upward rotation of the vermis has been suggested as a key indicator in differential diagnosis.

Recently, a new parameter for assessing the position of the vermis within the PF— called the vermian-crest angle (VCA)—was described using both three-dimensional ultrasound (3D-US) and prenatal magnetic resonance imaging (MRI) in a cohort of normal foetuses aged between 18 and 33 weeks of gestation, showing high reproducibility (Spinelli et al., 2019). Our previous study (Spinelli et al., 2019) demonstrated that the VCA is a stable parameter across different gestational ages (GAs) in a normal population. In contrast, vermian diameters measured using the same 3D multiplanar sonography technique demonstrate a linear correlation with gestational age, while vermian volume shows a quadratic correlation.

Furthermore, we retrospectively assessed the VCA in foetuses with abnormal PF on prenatal MRI and found that it increases proportionally with the degree of upward vermian rotation, showing significant enlargement in cases of DWM and BPC (Spinelli et al., 2019).

Collectively, these findings establish the reference framework underpinning the present investigation. In this study, we measured the VCA in foetuses with posterior fossa anomalies using 3D ultrasound, with the objectives of constructing a predictive model for differential diagnosis and corroborating our prior MRI-based results (Spinelli et al., 2019).

## Materials and methods

The study was initially conducted prospectively at our department and later extended to other centres in order to increase the number of cases investigated. We obtained 3D volumes from centres in different countries, which were retrospectively analysed. The recruitment period lasted 2 years. The study was conducted in accordance with the World Medical Association’s Declaration of Helsinki, and the protocol was approved by our institutional ethics committee.

### Study population

All patients who underwent prenatal US with evidence of an abnormal PF on cross-sectional imaging were included. An abnormal PF was defined as the presence of an enlarged cisterna magna (CM) on ultrasound. The inclusion criteria for the study are as follows: a CM measurement of ≥10 mm in the mid-sagittal plane or a clearly visible posterior fossa malformation (e.g., Dandy–Walker malformation, Blake’s pouch cyst, or vermian hypoplasia) on multiplanar 3D sonography. For all included patients, the following clinical data were recorded: gestational age (GA), associated anomalies, maternal age, singleton or multiple gestation, foetal karyotype (if available), prenatal MRI diagnosis (if available), GA at birth, mode of delivery, results of the neonatal examination, postnatal US and/or MRI diagnosis (if available), and—in cases of intrauterine or neonatal death or pregnancy termination—pathology reports (if available). Only cases with at least one postnatal diagnosis confirmed via US, MRI, and/or pathology report were included in the analysis.

### Measurements

The Voluson E10 or E8 (GE Healthcare Ultrasound, Zipf, Austria) ultrasound system, equipped with curved electronic matrix 4D (eM6C, RM6C) and transabdominal transducers (C2–9 MHz), was used. Following the routine 2D sonographic examination of the foetal brain, 3D volumes were acquired in a trans-cerebellar axial view during foetal and maternal rest, using a transabdominal acquisition angle of 55° to 65°, depending on GA. The 2D image was optimised before acquisition.

For multicentre cases, all contributing operators were experienced in foetal neurosonography and were instructed to acquire 3D brain volumes in standard multiplanar planes, specifically focusing on the posterior fossa. All datasets underwent a quality review before inclusion, and only those volumes that met predefined image quality criteria for VCA measurement were analysed.

All data were fully anonymised prior to analysis and stored on password-protected institutional servers, in accordance with national data protection regulations and institutional review board requirements.

The planes A, B, and C of the multiplanar images corresponded to the axial, coronal, and sagittal planes of the PF, respectively. Planes B (falx cerebri vertical) and C (falx cerebri horizontal) were adjusted to obtain an optimal midsagittal view of the brain in the A-plane, with the reference dot positioned in the middle of the vermis and the cervical spine vertically aligned. On the magnified A-plane, PF parameters were measured.

The VCA was measured as previously described (Spinelli et al., 2019), using two landmarks—the nodulus of the vermis, located at the level of the fastigial peak of the fourth ventricle, and the internal occipital crest, visible posterior to the cerebellar vermis as a hyperechoic line at its attachment to the falx cerebri. The VCA was defined by the convergence of two lines: the first tangent to the internal occipital crest and the second tangent to the nodulus of the vermis. All measurements were obtained using the ‘Dist. 2 Point’ and ‘Angle 2 Lines’ tools of the ultrasound machine, with callipers placed on the outer echogenic borders of the structures studied. PF contours were enhanced using the thin-slice (1–2 mm) volume contrast imaging (VCI) mode.

All measurements were compared with previously published reference values for normal cases (Spinelli et al., 2019). To assess the agreement between 3D-US and prenatal MRI, subgroups were compared with previously published MRI measurements (Spinelli et al., 2019).

For the main retrospective dataset, blinding of the measurers to the diagnosis was not possible, as the presence of pathology was part of the search criteria. However, during inter- and intra-observer analyses, all diagnoses were blinded to reduce measurement bias.

### Morphological severity grading

**Figure 1:**
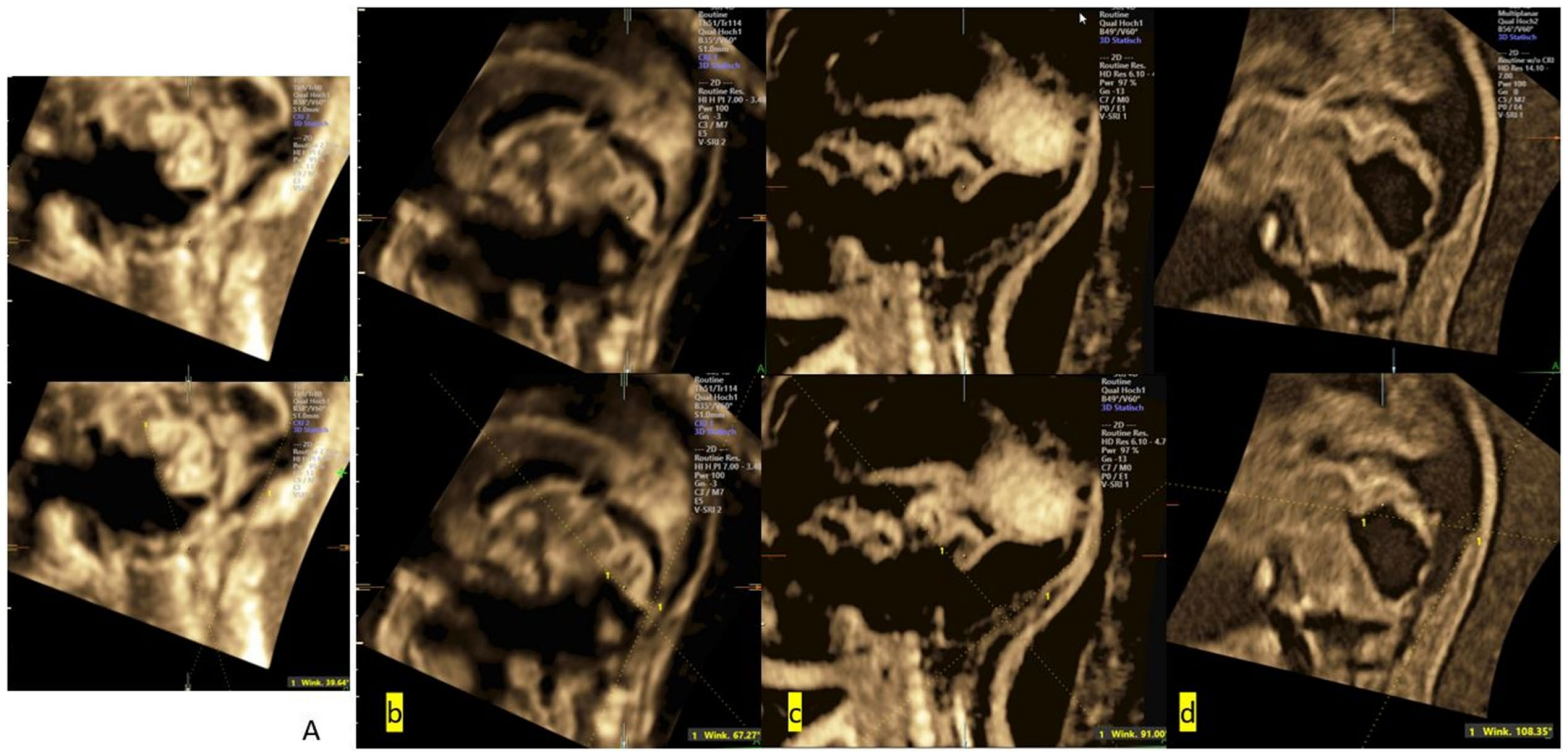
3D-US midsagittal view of the posterior fossa (PF) showing normal brain structure and abnormalities in the PF region: **(a)** a foetus at 25 weeks of GA with a normal PF (VCA = 39.64°); **(b)** a foetus at 24 weeks of GA with VH (VCA = 67.27°); **(c)** a foetus at 29 weeks of GA with BPC (VCA = 91.00°); and **(d)** a foetus at 30 weeks of GA with DWM (VCA = 108.35°). The VCA remained within the normal range in the cases with VH but changed in the cases with DWM, increasing with the severity of the pathological condition.

In this study, morphological severity was qualitatively graded based on 3D ultrasound imaging features. The grading considered the degree of upward rotation of the vermis, the size and configuration of the cisterna magna, and the presence of associated structural anomalies within the posterior fossa and supratentorial region. This classification was used solely for a descriptive correlation with VCA values and was not intended to predict postnatal functional outcomes (Figure 1).

### MRI use

This study relied exclusively on sonographic parameters. Prenatal or postnatal MRI, when available, served only as an ancillary diagnostic tool to corroborate the final diagnosis and was not employed for quantitative measurement or statistical comparison.

### Statistical analysis

Statistical analysis was performed using GraphPad Prism (version 8.2.1) (GraphPad Software, San Diego, CA) and SPSS version 19.0 (SPSS Inc., Chicago, IL, USA). Comparisons among the groups were performed using one-way ANOVA with Bonferroni adjustment, after verifying normal distribution with the Kolmogorov–Smirnov test. Tukey’s test was applied for multiple comparisons. Receiver operating characteristic (ROC) curves were used to describe the relationship between sensitivity and specificity across different VCA cutoff values for predicting PF malformations. Agreement between US and MRI was assessed using the intraclass correlation coefficient (ICC), with values <0.5, 0.5–0.75, 0.75–0.9, and >0.90 indicating poor, moderate, good, and excellent reliability, respectively (Spinelli et al., 2019). A *p*-value of < 0.05 was considered statistically significant. Given the retrospective and descriptive nature of this study, no *a priori* sample size calculation was performed. We therefore conducted a post-hoc power analysis and calculated the minimum detectable effect (MDE) for the main endpoints. Based on the observed group sizes (BPC *n* = 11, DWM *n* = 9, MCM *n* = 22, VH *n* = 11) and within-group variability, the omnibus one-way ANOVA yielded a Cohen’s f of 1.55, implying >99.9% power at *α* = 0.05 to detect the observed between-group effect. For the clinically most relevant contrast (BPC vs. DWM), the observed difference in VCA means (93.6° vs. 130.6°) with a pooled SD of 18.7° yielded a Cohen’s d of 1.98, providing >95% power (two-sided *α* = 0.05). Smaller contrasts (e.g., VH vs. MCM, d ≈ 0.25) were underpowered with the current sample size. As a sensitivity analysis, the MDE at 80% power and *α* = 0.05 was approximately f ≈ 0.33–0.35 for the omnibus ANOVA and d ≈ 1.26 for the BPC versus DWM comparison, both well below the observed effect sizes. These results support the adequacy of our sample for the primary objective—discriminating BPC from DWM and other PF anomalies—while recognising the limited power to detect small differences among milder categories.

## Results

During the study period, we included 53 foetuses with PF abnormalities confirmed postnatally: 11 with BPC, 9 with DWM, 11 with VH, and 22 with MCM. Table 1 summarises the clinical characteristics of the included cases. There were no significant differences between the groups in GA at diagnosis, maternal age, or the proportion of singleton versus multiple pregnancies. Associated anomalies were more frequently observed in the DWM group.

**Table 1:** Clinical characteristics of the included cases.

Characteristics	Value
Age, y	33 (range 26–45)
Gestational age, weeks	24.5 ± 5.43
Body mass index, kg/m2	23 ± 3.4
Gravidity	1.7 ± 0.58
Parity	1.1 ± 0.39
Karyotype analysis	5* abnormal /22 analysed
Associated malformations	*N* = 25**
Intrauterine foetal deaths	*N* = 1***
Termination of pregnancy	*N* = 7
Male/female ratio	0.6

*One trisomy 18, one trisomy 13, one triploid, one deletion on chromosome 4, and one deletion on chromosome 1. Two of the five karyotypic anomalies were found in foetuses with DWM, two were associated with MCM, and one with BPC.

**Cardiac (*n* = 6), brain (*n* = 14), skeletal (*n* = 5), renal (*n* = 1), gastrointestinal (*n* = 1), genital (*n* = 2), single umbilical artery (SUA).

***1 with isolated DWM.

Table 2 summarises the VCA measurements for each diagnostic category. In the cases with MCM (mean VCA: 66.79° ± 12.98) and VH (mean VCA: 70.02° ± 11.99), the values did not differ significantly from the reference range established in our previous normative study (*p* = 0.84 and *p* = 0.95, respectively). In contrast, VCA values were significantly increased in the cases with BPC (mean VCA: 93.58° ± 20.09, *p* ≤ 0.05) and markedly elevated in those with DWM (mean VCA: 130.59° ± 16.75, *p* ≤ 0.01). These differences were confirmed by univariate ANOVA followed by Tukey’s post-hoc comparisons.

**Table 2:** Summary of VCA measurements by diagnosis.

Diagnosis	*n*	Mean VCA (°)	SD	Comparison with reference range	*P*-value
Mega Cisterna Magna (MCM)	22	66.79	12.98	Not different	0.84
Vermian Hypoplasia (VH)	11	70.02	11.99	Not different	0.95
Blake’s Pouch Cyst (BPC)	11	93.58	20.09	Increased	≤0.05
Dandy–Walker Malformation (DWM)	9	130.59	16.75	Increased	≤0.01

Values are expressed as mean ± standard deviation (SD) and range. *P*-values refer to Tukey’s *post-hoc* comparisons with the control population following univariate ANOVA.

**Figure 2:**
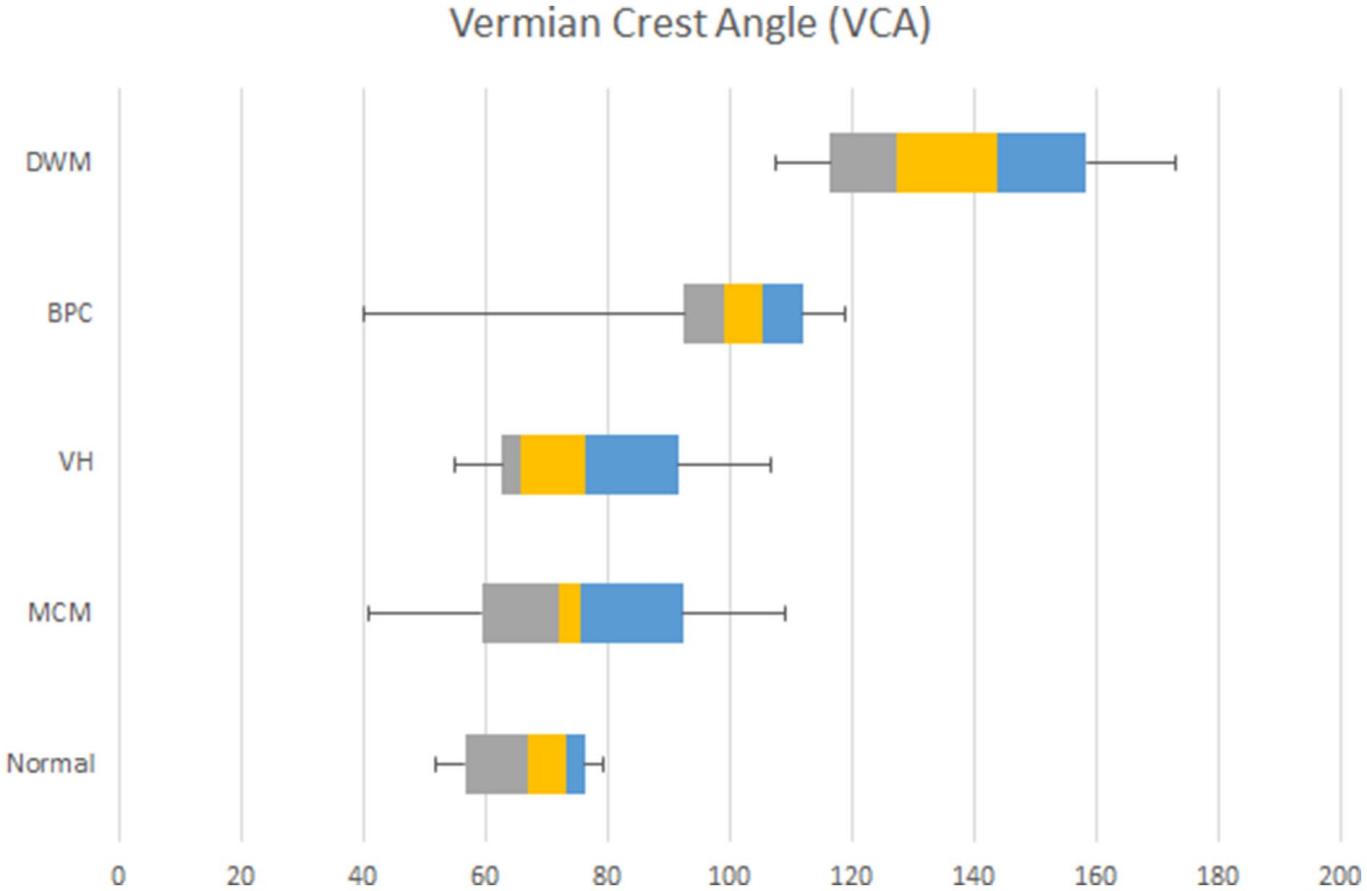
Box-and-whisker plot showing the distribution of the VCA in normal^10^ foetuses and in those with MCM, VH, BPC, and DWM. Medians are indicated by the line inside each box, the 25th and 75th percentiles by the box limits and the 5th and 95th percentiles by the lower and upper bars. VCA increased significantly (*) in both BPC and DWM compared to the normal reference range^10^.

**Figure 3:**
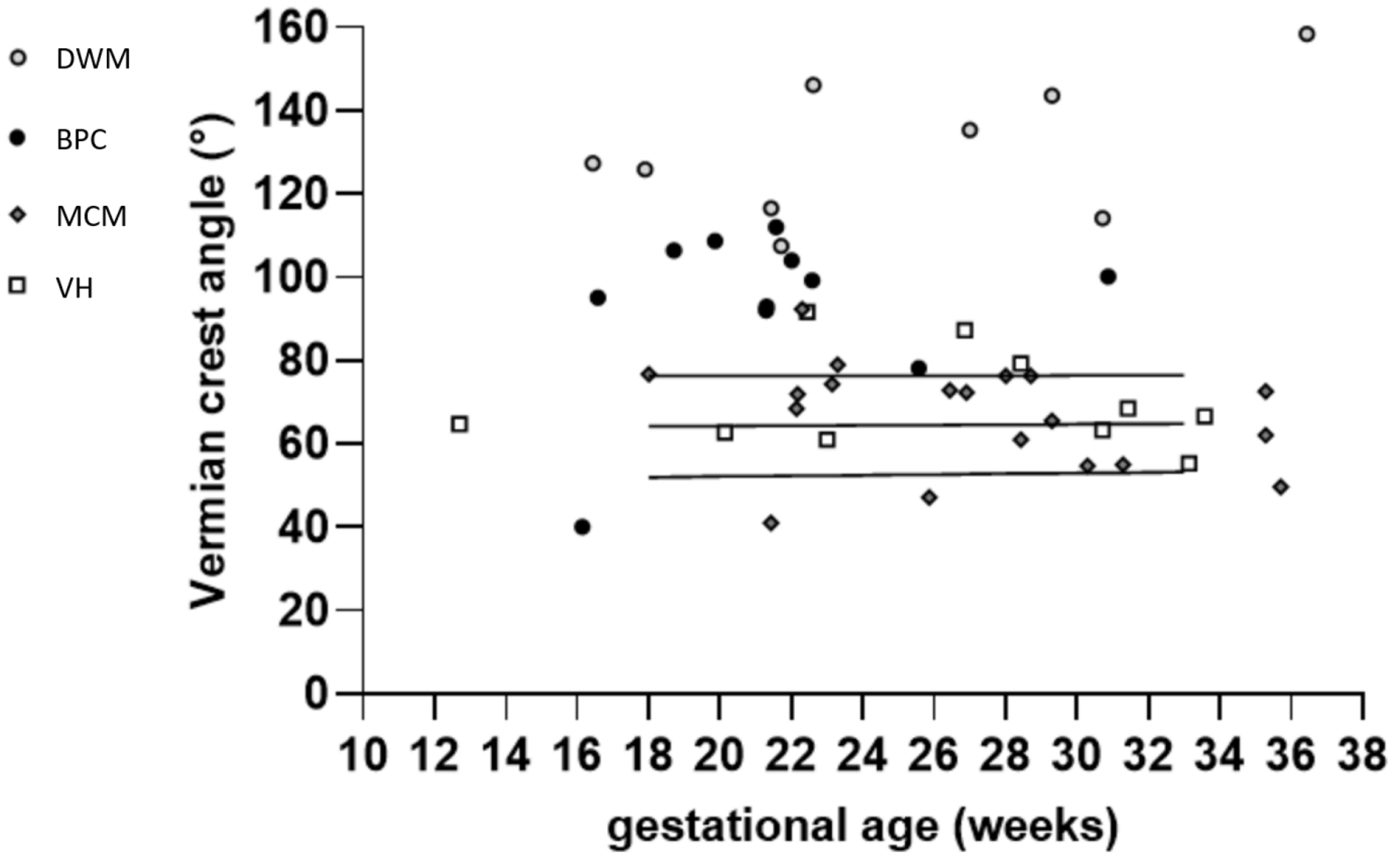
Measurements of the VCA in the subgroups of foetuses with a pathological PF, shown on a plot displaying the observed VCA measurements and the fitted 10th, 50th, and 90th percentiles for GA from a previous study (black continuous lines)^10^.

**Figure 4:**
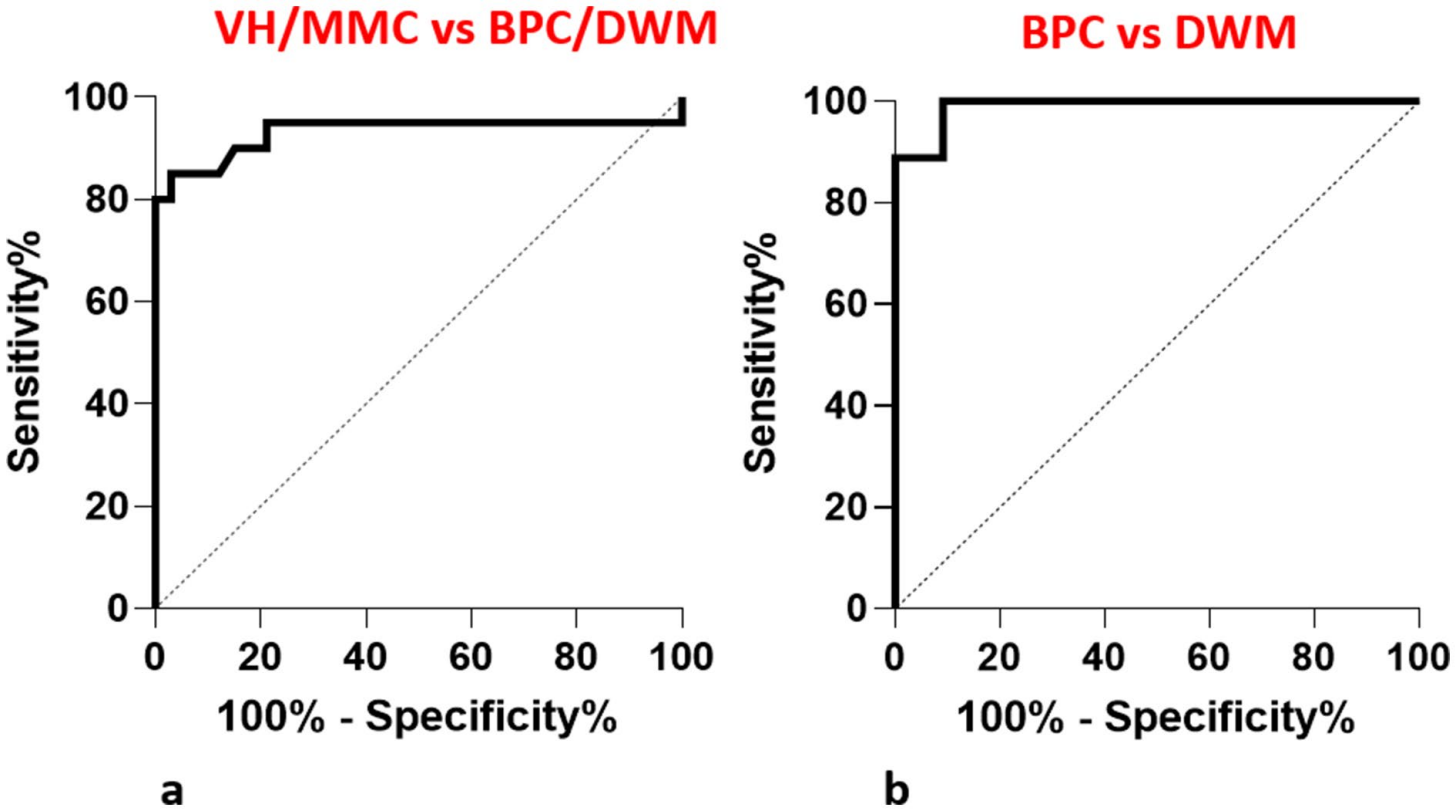
ROC analyses assessing the diagnostic performance of the VCA. **(a)** The area under the ROC curve for distinguishing VH or MCM from BPC or DWM was 0.93 (95% CI 0.83–1). **(b)** The area under the ROC curve for distinguishing BPC from DWM was 0.99 (95% CI 0.96–1.00).

Figure 2 depicts the box and whisker plots of the distribution of the VCA in normal and pathological cases. Figure 3 shows a plot of the measurements of the VCA in pathological cases on the scatterplots showing the distribution of VCA in normal foetuses against GA, of a previous paper (Spinelli et al., 2019). Figure 4 presents ROC analyses assessing the diagnostic performance of the VCA. The area under the ROC curve (AUC) for discriminating VH or MCM from BPC or DWM was 0.93 (95% CI 0.83–1; *p* < 0.0001). A VCA cutoff value of 80.1° yielded a sensitivity and specificity of 90% (95%CI 69.90 to 98.22%) and 84.85% (95%CI 69.08 to 93.35%), respectively (Figure 4 a). The AUC for discriminating the BPC group from the DWM group was 0.99 (95% CI 0.96–1.00; *p* < 0.0002). At a VCA cutoff value of 107°, the sensitivity and specificity were 100% (95%CI 70.09 to 100%) and 90.91% (95%CI 62.26 to 99.53%), respectively (Figure 4 b).

**Figure 5:**
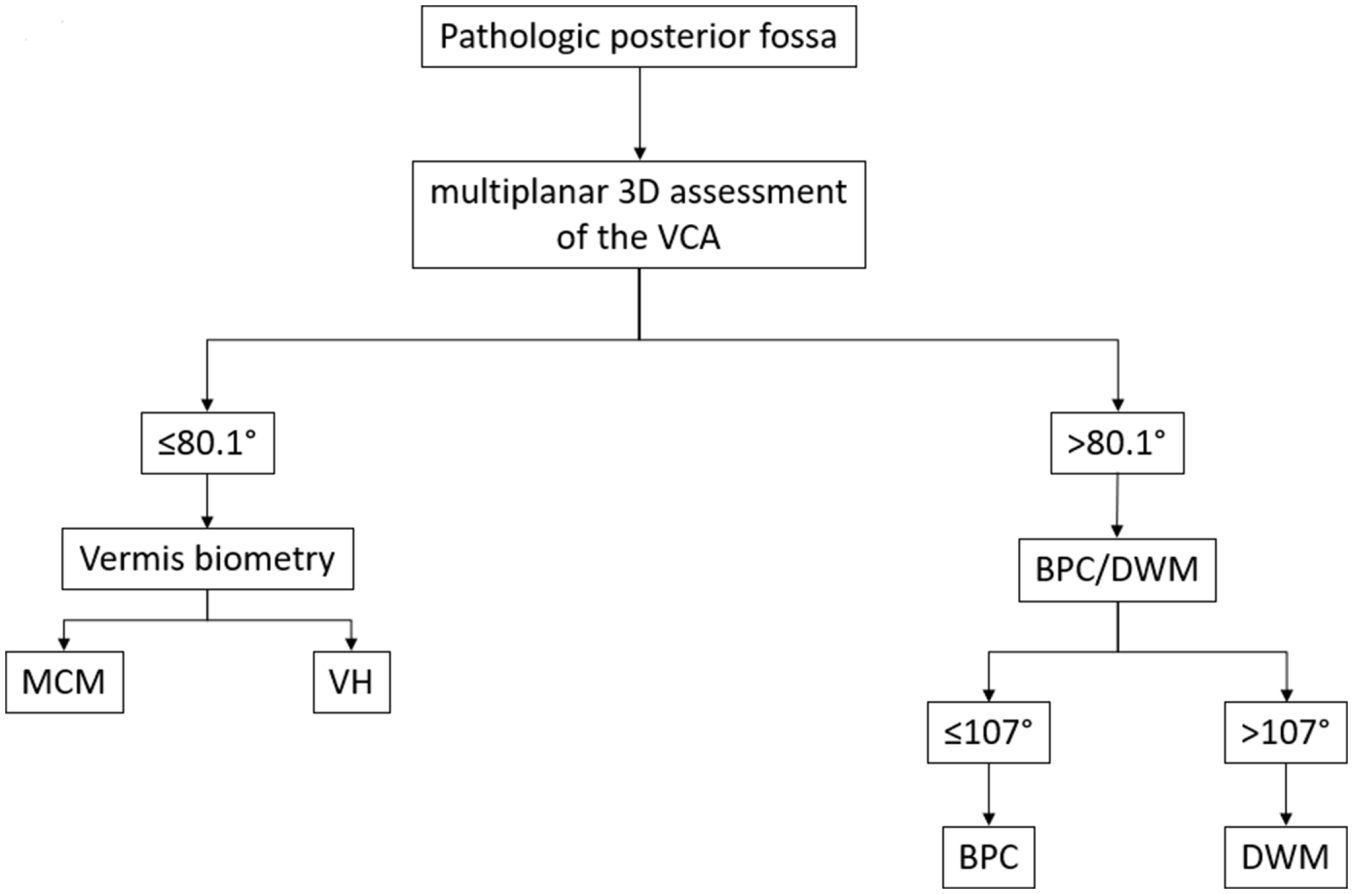
Proposed diagnostic flowchart for the classification of foetal posterior fossa anomalies using the Vermian-Crest Angle (VCA). The algorithm begins with the identification of a pathologic posterior fossa on prenatal imaging. (1) A multiplanar 3D assessment of the VCA is performed. (2) If the VCA is ≤80.1°, the next step is to evaluate vermian biometry. Normal vermian size suggests MCM. (4) Reduced vermian size suggests VH. (5) If the VCA is >80.1°, the anomaly is likely either a BPC or a DWM. (6) In these cases, a second VCA threshold (107°) is applied. (7) ≤107° supports the diagnosis of BPC. (8) >107° supports the diagnosis of DWM. This decision tree is based on the cutoff values derived from the ROC curve analysis in the present study, integrating the VCA with vermian biometry to improve the differentiation between common cystic posterior fossa anomalies.

Based on these cutoff values, we developed a diagnostic flowchart to guide the differentiation of common cystic posterior fossa anomalies in the prenatal setting (Figure 5). The algorithm begins with the identification of a posterior fossa anomaly on prenatal imaging, followed by multiplanar 3D assessment of the VCA. A VCA of ≤80.1° suggests either MCM or VH. These anomalies can be distinguished by evaluating vermian biometry, with the normal vermian size indicating MCM and the reduced vermian size indicating VH. A VCA > 80.1° suggests either BPC or DWM. In these cases, applying a second cutoff at 107° allows further differentiation: values ≤107° support the diagnosis of BPC, whereas values >107° are consistent with DWM. This two-step approach, integrating VCA thresholds with vermian biometry, provides a simple and reproducible method to improve diagnostic accuracy in clinical practice.

By comparing subgroups with the respective published MRI data (Spinelli et al., 2019), we found good agreement between 3D-US and MRI measurements (ICC = 0.71) (95% CI 0.55–0.87).

### Structured discussion/comment

Our results demonstrate clear differences in VCA measurements among the various posterior fossa anomalies, with DWM and BPC showing significantly higher values compared to MCM and VH. These differences were also reflected in the ROC curve analysis, which identified clinically relevant cutoff values with high sensitivity and specificity. This finding supports the potential of the VCA as a diagnostic tool in the prenatal setting.

### Main findings

In this study, we evaluated the VCA in foetuses with posterior fossa anomalies using 3D ultrasound, and we developed a predictive model for differential diagnosis. Our findings confirm previous MRI-based results, showing that the VCA increases in proportion to the degree of upward rotation of the vermis and is significantly higher in DWM and BPC compared to MCM and VH.

The clinical importance of these findings lies in the ability to differentiate more severe anomalies, such as DWM, from milder conditions (e.g., MCM) during prenatal assessment. In our cohort, the VCA tended to increase in parallel with the morphological severity of the posterior fossa anomaly, as graded qualitatively according to imaging features. Specifically, the cases showing greater upward rotation of the vermis, a larger or abnormally configured cisterna magna, and additional structural anomalies had the highest VCA values. This observation is consistent with previous reports linking more pronounced morphological alterations to a higher likelihood of associated anomalies and a less favourable clinical course.

This is particularly relevant because the prenatal diagnosis of PF anomalies often shares overlapping imaging features, making prognosis and counselling challenging. The VCA provides an additional, reproducible parameter that can be integrated with existing diagnostic criteria, including vermis biometry, cerebellar anatomy and morphometry, to improve diagnostic accuracy.

### Measurement reproducibility

Our data show that the VCA is a stable parameter across gestational ages, in line with previous reports in normal foetuses. This stability is a strength for clinical application, as it enables comparisons without the need for GA adjustment. The high inter- and intra-observer agreement within our team suggests that VCA measurement is robust when performed by experienced operators. Although we did not assess inter-operator variability across all contributing centres, the standardised acquisition protocol and rigorous image quality selection likely contributed to measurement consistency.

### Sample size consideration

The rarity of these pathologies inevitably limits the sample size. Based on the current literature on prenatal ultrasound studies of the posterior fossa, the reported case series range from single case reports to a maximum of approximately 90 cases (Volpe et al., 2021). Our cohort size was therefore within the expected range; however, the small number of cases within each diagnostic subgroup may have limited the power to detect smaller differences between groups.

This limitation was partly mitigated by our post-hoc power analysis, which confirmed sufficient power for the primary objective of differentiating DWM and BPC from other anomalies but indicated insufficient power for detecting subtle contrasts between MCM and VH.

### MRI correlation

Our study was based solely on sonographic data. Although some cases also underwent foetal MRI, the inclusion of MRI as a requirement would have further reduced the sample size. Nevertheless, our results are consistent with our previous MRI-based study and support the role of the VCA as a meaningful diagnostic marker. Only a prospective, well-structured multicentre study with systematic MRI correlation could definitively address the remaining uncertainties.

### Blinding consideration

The retrospective design limited blinding to the diagnosis during the initial measurements; however, blinding was applied during the inter- and intra-observer variability analyses, reducing potential measurement bias.

## Conclusion

In conclusion, the VCA is a simple and reproducible measurement that enhances the prenatal differential diagnosis of PF anomalies. When combined with other established sonographic parameters, it can aid in stratifying cases into those that require closer follow-up and multidisciplinary counselling and those that are likely to have a more favourable prognosis.

## Data Availability

The raw data supporting the conclusions of this article are available by request.

## References

[ref1] Altmann R. Schertler C. Scharnreitner I. Arzt W. Dertinger S. Scheier M. (2020). Diagnosis of foetal posterior fossa malformations in high-risk pregnancies at 12-14 gestational weeks by transvaginal ultrasound examination. Fetal Diagn. Ther. 47, 182–187. doi: 10.1159/000501500, PMID: 31311012

[ref2] Barkovich A. J. Millen K. J. Dobyns W. B. (2009). A developmental and genetic classification for midbrain-hindbrain malformations. Brain 132, 3199–3230. doi: 10.1093/brain/awp247, PMID: 19933510 PMC2792369

[ref3] Barzegar M. Malaki M. Sadegi-Hokmabadi E. (2013). Joubert syndrome with variable features: presentation of two cases. Iran. J. Child Neurol. 7, 43–46. 24665296 PMC3943034

[ref4] Dekain L. H. Kanel H. El-Bashir H. O. (2014). Joubert syndrome labeled as hypotonic cerebral palsy. Neurosciences (Riyadh) 19, 233–235. 24983287 PMC4727659

[ref5] Kapur R. P. Mahony B. S. Finch L. Siebert J. R. (2009). Normal and abnormal anatomy of the cerebellar vermis in midgestational human foetuses. Birth Defects Res. A Clin. Mol. Teratol. 85, 700–709. doi: 10.1002/bdra.20589. 19441098

[ref6] Katorza E. Bertucci E. Perlman S. Taschini S. Ber R. Gilboa Y. . (2016). Development of the foetal vermis: new biometry reference data and comparison of 3 diagnostic modalities–3D ultrasound, 2D ultrasound, and MRI maging. AJNR Am. J. Neuroradiol. 37, 1359–1366. doi: 10.3174/ajnr.A4725, PMID: 27032974 PMC7960333

[ref7] Lei T. Xie H. N. Zhu Y. X. Zheng J. Zhang F. Feng J. L. . (2015). Date-independent parameters: an innovative method to assess foetal cerebellar vermis. Cerebellum 14, 231–239. 25577030 10.1007/s12311-014-0632-x

[ref8] Limperopoulos C. Robertson R. L. Estroff J. A. Barnewolt C. Levine D. Bassan H. . (2006). Diagnosis of inferior vermian hypoplasia by foetal MRI: potential pitfalls and neurodevelopmental outcome. Am. J. Obstet. Gynecol. 194, 1070–1076. doi: 10.1016/j.ajog.2005.10.191, PMID: 16580298 PMC1557637

[ref9] Malinger G. Ginath S. Sagie-Lerman T. Watemberg N. Lev D. Glezerman M. . (2001). The foetal cerebellar vermis: normal development as shown by transvaginal ultrasound. Prenat. Diagn. 21, 687–692. doi: 10.1002/pd.137 11536272

[ref10] Milani H. J. F. Barreto E. Q. S. Ximenes R. L. D. S. Baldo C. A. R. Araujo Júnior E. Moron A. F. . (2019). Foetal posterior fossa malformations: review of the current knowledge. Radiol. Bras. 52, 380–386. doi: 10.1590/0100-3984.2018.0141 32047332 PMC7007051

[ref11] Paladini D. Donarini G. Parodi S. Volpe G. Sglavo G. Fulcheri E. (2019). Hindbrain morphometry and choroid plexus position in differential diagnosis of posterior fossa cystic malformations. Ultrasound Obstet. Gynecol. 54, 207–214. doi: 10.1002/uog.20120, PMID: 30207001

[ref12] Pertl B. Eder S. Stern C. Verheyen S. (2019). The “vermian-crest angle”: does it allow accurate categorisation of fetal upward rotation of cerebellar vermis on intrauterine MRI? A pilot study. Ultraschall Med. 40, 692–721. 30954236 10.1016/j.crad.2019.02.017

[ref13] Poretti A. Boltshauser E. Doherty D. (2014). Cerebellar hypoplasia: differential diagnosis and diagnostic approach. Am. J. Med. Genet. Semin. Med. Genet., 1, 211–226. doi: 10.1002/ajmg.c.31398 24839100

[ref14] Rizzo G. Pietrolucci M. E. Mammarella S. Dijmeli E. Bosi C. Arduini D. . (2012). Assessment of cerebellar vermis biometry at 18-32 weeks of gestation by three-dimensional ultrasound examination. J Maternal-Foetal Neonatal Med. 25, 519–522. doi: 10.3109/14767058.2011.622006, PMID: 21919549

[ref15] Robinson A. J. (2014). Editorial. Inferior vermian hypoplasia – preconception, misconception. Ultrasound Obstet. Gynecol. 43, 123–1366. doi: 10.1002/uog.13296 24497418

[ref16] Robinson A. J. Blaser S. Toi A. Chitayat D. Halliday W. Pantazi S. . (2007). The foetal cerebellar vermis: assessment for abnormal development by ultrasonography and magnetic resonance imaging. Ultrasound Q. 23, 211–223. doi: 10.1097/RUQ.0b013e31814b162c, PMID: 17805192

[ref17] Spinelli M. Di Meglio L. Mosimann B. Di Naro E. Surbek D. Raio L. . (2019). The Vermian-crest angle: a new method to assess Foetal vermis position within the posterior Fossa using 3-dimensional multiplanar sonography. Foetal Diagn Ther. 46, 223–230. doi: 10.1159/000494721, PMID: 30517923

[ref18] Spinelli M. Wiest R. Di Meglio L. Baumann M. Raio L. Surbek D. . (2019). The "vermian-crest angle": does it allow accurate categorisation of foetal upward rotation of cerebellar vermis on intrauterine MRI? A pilot study. Clin Radiol. 74, 489.e1–489.e7. doi: 10.1016/j.crad.2019.02.017, PMID: 30954236

[ref19] Sun L. Guo C. Yao L. Zhang T. Wang J. Wang L. . (2019). Quantitative diagnostic advantages of three-dimensional ultrasound volume imaging for foetal posterior fossa anomalies: preliminary establishment of a prediction model. Prenat. Diagn. 39, 1086–1095. doi: 10.1002/pd.5549, PMID: 31441071

[ref20] ten Donkelaar H. J. Lammens M. Hori A. (2014). Clinical Neuroembryology: Development and developmental disorders of the human central nervous system. 2nd Edn. Berlin, Heidelberg: Springer.

[ref21] Tilea B. Delezoide A. L. Khung-Savatovski S. Guimiot F. Vuillard E. Oury J. F. . (2007). Comparison between magnetic resonance imaging and fetopathology in the evaluation of foetal posterior fossa non-cystic abnormalities. Ultrasound Obstet. Gynecol. 29, 651–659. doi: 10.1002/uog.4012, PMID: 17476704

[ref22] Vatansever D. Kyriakopoulou V. Allsop J. M. Fox M. Chew A. Hajnal J. V. . (2013). Multidimensional analysis of foetal posterior fossa in health and disease. Cerebellum 12, 632–644. doi: 10.1007/s12311-013-0470-2 23553467

[ref23] Viñals F. Muñoz M. Naveas R. Shalper J. Giuliano A. (2005). The foetal cerebellar vermis: anatomy and biometric assessment using volume contrast imaging in the C-plane (VCI-C). Ultrasound Obstet. Gynecol. 26, 622–627. doi: 10.1002/uog.2606 16254881

[ref24] Volpe P. De Robertis V. Volpe G. Boito S. Fanelli T. Olivieri C. . (2021). The position of the choroid plexus of the fourth ventricle in the first- and second-trimester foetuses: an early different approach to diagnostic imaging of cystic posterior fossa anomalies. Ultrasound Obstet. Gynecol. 58, 568–575. doi: 10.1002/uog.23651, PMID: 33847428

[ref25] Xie J. X. You J. H. Chen X..K. Su Y-M. Liu J-R. Su S-S. . Three-dimensional sonographic minute structure analysis of foetal cerebellar vermis development and malformations: utilizing volume contrast imaging. J. Med. Ultrason. (2001). 209;46(1):113–122. 10.1007/s10396-018-0906-x 30291575

[ref26] Zalel Y. Gilboa Y. Gabis L. Ben-Sira L. Hoffman C. Wiener Y. . (2006). Rotation of the vermis as a cause of enlarged cisterna magna on prenatal imaging. Ultrasound Obstet. Gynecol. 27, 490–493. doi: 10.1002/uog.2768, PMID: 16619381

[ref27] Zalel Y. Seidman D. S. Brand N. Lipitz S. Achiron R. (2002). The development of the foetal vermis: an in utero sonographic evaluation. Ultrasound Obstet. Gynecol. 19, 136–139. doi: 10.1046/j.0960-7692.2001.00621.x. 11876804

