# Correction: The vermian-crest angle: a prediction model for foetal cystic posterior fossa anomalies

**DOI:** 10.3389/fnins.2026.1858645

**Published:** 2026-04-29

**Authors:** Frontiers Production Office

**Affiliations:** Frontiers Media SA, Lausanne, Switzerland

**Keywords:** vermian-crest angle, posterior fossa, vermis, cerebellum, prenatal diagnosis, three-dimensional ultrasound scan, Blake's pouch cyst, Dandy–Walker malformation

The reference for Limperopoulos and du Plessis, 2006 was erroneously written as “Limperopoulos C., du Plessis A. J. (2006). Diagnosis of inferior vermian hypoplasia by foetal MRI: potential pitfalls and neurodevelopmental outcome. *Am. J. Obstet. Gynecol*.194, 1070–1076. doi: 10.1097/MOP.0b013e32801080e8”. It should be “Limperopoulos, C., Robertson, R. L., Estroff, J.A., Barnewolt, C., Levine, D., Bassan, H., et al. (2006). Diagnosis of inferior vermian hypoplasia by foetal MRI: potential pitfalls and neurodevelopmental outcome. *Am. J. Obstet. Gynecol*.194, 1070–1076. doi: 10.1016/j.ajog.2005.10.191”.

The reference for Poretti et al., 2014 was erroneously written as “Poretti A., Boltshauser E., Doherty D. (2014). Cerebellar hypoplasia: differential diagnosis and diagnostic approach. Am. J. Med. Genet. Semin. Med. Genet., 1:211–226. doi: 10.1148/rg.351140038”. It should be “Poretti, A., Boltshauser, E., and Doherty, D. (2014). Cerebellar hypoplasia: differential diagnosis and diagnostic approach. *Am. J. Med. Genet. Semin. Med. Genet*. 1, 211–226. doi: 10.1002/ajmg.c.31398”.

The original version of this article has been updated.

